# Comparison of biofilm formation and migration of Streptococcus mutans on tooth roots and titanium miniscrews

**DOI:** 10.1002/cre2.101

**Published:** 2018-02-21

**Authors:** Kittipong Laosuwan, Don Jeevanie Epasinghe, Zhaoming Wu, Wai Keung Leung, David William Green, Han Sung Jung

**Affiliations:** ^1^ Applied Oral sciences, Faculty of Dentistry The University of Hong Kong Hong Kong; ^2^ Periodontology, Faculty of Dentistry The University of Hong Kong Hong Kong; ^3^ Dept of Oral Biology Yonsei University College of Dentistry Korea

## Abstract

Periodontitis and peri‐implantitis are inflammatory diseases caused by periodontal pathogenic bacteria leading to destruction of supporting periodontal/peri‐implant tissue. However, the progression of inflammatory process of these two diseases is different. The bacterial biofilm is the source of bacteria during the inflammatory process. As the bacteria migrate down the surface of tooth or titanium implant, the inflammation spreads along with it. Streptococcus mutans has an important role in oral bacterial biofilm formation in early stage biofilm before the microbiota shift to late stage and become more virulent. The other major difference is the existence of periodontal ligament (PDL) cells in normal teeth but not in peri‐implant tissue. This study aims to compare the S. mutans bacterial biofilm formation and migration on 2 different surfaces, tooth root and titanium miniscrew. The biofilm was grown with a flow cells system to imitate the oral dynamic system with PDL cells. The migration distances were measured, and the biofilm morphology was observed. Data showed that the biofilm formation on miniscrew was slower than those on tooth root at 24 hr. However, there were no difference in the morphology of the biofilm formed on the tooth root with those formed on the miniscrew at both 24 and 48 hr. The biofilm migration rate was significantly faster on miniscrew surface compare with those on tooth root when observe at 48 hr (p < .001). There are no significant differences in biofilm migration within miniscrew group and tooth root group despite the exiting of PDL cell (p > .05). The biofilm's migration rate differences on various surfaces could be one of the factors accounting for the different inflammatory progression between periodontitis and peri‐implantitis disease.

## 1 INTRODUCTION

Periodontitis and peri‐implantitis are the most common infectious inflammatory diseases of tooth sockets and gum tissues including around the tissue surrounding bases of teeth or implants that leads to massive tissue resorption. Microbial overgrowth around compromised tooth sockets triggers exorbitant host inflammation. Both types of conditions have identical origins, but the pathogenic pathways are different between the natural teeth versus the artificial implant due to various factors (Berechet, Ionaşcu, Sîrbu, & Sîrbu, 2013; Berglundh, Zitzmann, & Donati, 2011).

Several studies reveal that the inflammatory cell infiltration in periodontitis and peri‐implantitis infected tissue are similar, dominated by B‐cells and plasma cells (Dhir, Mahesh, Kurtzman, & Vandana, 2013). However, the dynamic of infectivity and pathogenesis are thought to be different because natural teeth are surrounded by supracrestal tight connective tissue fibers, which prevent the inflammatory lesion from expanding into surrounding bone (Dhir et al., 2013). This could explain the larger bone resorption around implants. Moreover, peri‐implantitis has no connective tissue buffering to protect inflammatory lesions from spreading; thus, lesions often spread and expand to surrounding bone marrow rapidly and more aggressively (Berechet et al., 2013). In vivo studies on inflammatory cell infiltration also reveal that during inflammation of the peri‐implant tissues and periodontal tissue, the inflammatory cells infiltrate more apically towards the peri‐implant mucosa than in the periodontal tissue or gingiva around the natural tooth. This is due to peri‐implant tissue has a less protective capacity than periodontal tissue in terms of lower tissues buffering, healing, and regeneration (Berechet et al., 2013; Berglundh et al., 2011; Dhir et al., 2013).

The gingival mucosa is the first barrier that preventing the possibility of bacterial invasion from the oral cavity into the underlying periodontal and peri‐implant tissues. Both periodontal and peri‐implant mucosa are composed of highly keratinized epithelium, sulcular epithelium, and junctional epithelium along the underlying connective tissue. There are clear differences in the junctional proteins between implant and epithelium and with basal lamina junctions at the interface between natural tooth and periodontal tissue (Dhir et al., 2013). Fiber‐enriched connective tissues are observed in peri‐implant mucosa with close contact to with the titanium surface that generates a tight barrier to seal off the bone tissue surrounding the implant from the external environment in oral cavity and also prevents the infiltration of microbial pathogens. These small details can explain the strong differences in pathogenesis. (Berechet et al., 2013; Dhir et al., 2013).

In natural teeth, junctional epithelium forms and attaches along the entire length of the dentine perimeter and enamel by hemidesmosome and basal laminar. However, around the peri‐implant, the junctional epithelium is generated only at the apical regions. Consequently, the mechano‐protective barrier of peri‐implant mucosa is not so tightly engaged to the implant surface as periodontal mucosa, thus making it more vulnerable to the penetration of bacterial infiltration and biofilm formation much deeper into the root part and socket (Berechet et al., 2013; Dhir et al., 2013; Palomo & Terézhalmy, 2014).

Bacterial biofilm covering infected teeth and implant is a major source of bacteria during the inflammatory process (Hasan & Palmer, 2014). Several studies investigating bacterial biofilm that forms on the surfaces of materials, cobalt–chromium, titanium, dentin, and hydroxyapatite and all have different binding affinities to a range of bacteria (Patel et al., 2016; Yoshida, Imai, Hanada, & Hayakawa, 2013). Both natural teeth and titanium implants have a binding affinity to bacterial biofilms. This begins with salivary proteins pellicles adsorb to the tooth and implant surface, followed by initial colonization, and maturation of biofilms. As the biofilm expands and matures, its virulence increases. Surface properties of implants, chemical composition, roughness, free energy, surface topography and surface stiffness, and so on have direct influence on the nature of bacteria colonization and biofilm formation (Busscher, Rinastiti, Siswomihardjo, & Van der Mei, 2010; Dhir, 2013; Souza et al., 2016; Subramani, Jung, Molenberg, & Hämmerle, 2009). Periodontal biofilm microbiota has been thoroughly investigated and characterized, and *Streptococcus mutans* (*S*. *mutans*) has been found to be an important microorganism during initial biofilm formation (Koo, Xiao, Klein, & Jeon, 2010).

*Streptococcus mutans* is a gram‐positive coccus colonizing on the supragingival region. It is the bacteria in the primary bacterial species associated with the early stage of bacterial biofilm formation (Koo et al., 2010). In addition, recent studies found that *S*. *mutans* is located in the subgingival biofilm collected from both patients with healthy gingival condition and with periodontal disease. However, the behavior of the bacteria, such as sucrose consumption, acid, and glycan production, was different (Dani et al., 2016). *S*. *mutans* expresses glucosyltransferases (Gtfs), which translates sucrose molecules into glucan during biofilm formation (Koo et al., 2010).

There are three subtypes of glucan molecules produced by specific Gtfs: water‐soluble glucan, semiwater‐soluble glucan, and water‐insoluble glucan. *S*. *mutans* predominantly produces water‐insoluble glucan, which is highly adhesive to surfaces inside the oral cavity. The water insoluble properties greatly contribute to biofilm formation in the dynamic oral microenvironment. Removal of *S*. *mutans* from the microorganism ecosystem dramatically affects biofilm formation in the oral cavity leading to large reduction in biofilm volume and slows the biofilm development. Glucans produced by *S*. *mutans* also provide binding sites for the latecomer bacteria, which lack ability to attach to the oral surface. As the biofilm matures, the proportion of *S*. *mutans* decreases and the microbiota shifts to late colonizer bacterial assemblages (Koo et al., 2010; Mattos‐Graner, Smith, King, & Mayer, 2000).

Although the nature of bacterial biofilm migration at solid surfaces in the oral cavity has not been satisfactorily explained, it is likely to contribute to the spread of infection and inflammation in soft mucosa and may in part account for the differences between periodontal biofilm and peri‐implant biofilm formation. This study aimed to investigate the differential formation and migration of *S*. *mutans* bacterial biofilms on the surface of titanium miniscrews (used as a substitute for titanium implants) versus tooth root surfaces using a novel flow system model. In addition, this study shall explore the possibility that periodontal ligament (PDL) cells have an effect in the bacterial biofilm formation and its migration over tooth and titanium miniscrew implant surfaces.

## 2 MATERIAL AND METHODS

### 2.1 Miniscrews, tooth roots, bacteria strains, and cell culture condition

Pure titanium miniscrews (9 mm length) without a surface coating were provided by the Yonsei University College of Dentistry in Seoul, Korea. Fifty of natural tooth roots were collected from extracted upper molar teeth of patient due to extensive carious lesion from the Prince Philip Dental Hospital, Hong Kong. Ethical approval and patient consent were made before collecting teeth for this study. The roots were cut and separated from the crown and had a length of 9 mm from apical tip same as the miniscrews length.

*Streptococcus mutans* cultures were grown anaerobically on a brain heart infusion (BHI: ThermoFisher SCIENTIFIC Ltd., Waltham, USA) agar plates at 37 °C (Koo et al., 2010). Immortalized human periodontal ligament cells (ihPDL cells) were grown on a 10‐cm culture dishes with high‐glucose Dulbecco's Modified Eagle Medium (ThermoFisher SCIENTIFIC Ltd., Waltham, USA) and supplemented with 1% penicillin/streptomycin (P/S) and 10% fetal bovine serum at 37 °C until the cells were confluent (Marchesan, Scanlon, Soehren, Matsuo, & Kapila, 2011).

### 2.2 Soft agar gel assay

Soft agar gel was used to culture ihPDL cells and to hold the miniscrew and tooth root in a fixed upright position during the biofilm inoculation and growth phase. The ihPDL cells were trypsinized from the 10‐cm culture dish and resuspended, and cell concentration was adjusted to 2 ml at 5 × 10^9^ (Dhir, 2013) cells/ml in media. A block of agarose gel (0.7%) was melted and cooled to 37 °C in a water bath. Next, 2 ml of agarose gel was pipetted into 2 ml of the ihPDL cell suspension (leading to a final cell concentration of 2.5 × 10^9^; Dhir, 2013), with gentling mixing before rapid pipetting 1.6 ml of gel/cell suspension into the lower chamber of a 6‐well gradient culture container (MINUCELLS and MINUTISSUE, Bad Abbach*,* German). The gel was left to set for 30 min (Horibata, Vo, Subramanian, & Thompson, 2015). The miniscrew/tooth root were placed in upright position at the center of soft agar gel prepared in the previous step (control with soft agar gel without cells) and the container were then closed and locked.

### 2.3 Growing *S*. *mutans* biofilm on miniscrews and tooth roots using the flow system

*Streptococcus mutans* was collected from the BHI agar plate and resuspended in BHI medium (ThermoFisher Scientific Ltd., Waltham, USA). Subsequently, the concentration was adjusted to 1 × 10^9^ (Dhir, 2013) colony‐forming unit/ml. The full inoculation was performed by injecting the bacteria suspension into the upper chamber of a gradient culture container and was then incubated anaerobically at 37 °C for 2 hr.

**Figure 1:**
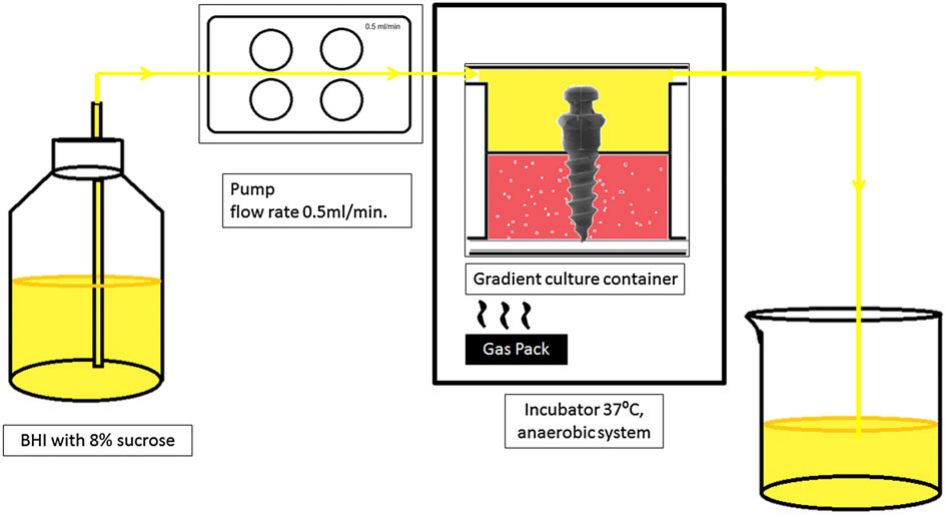
Proposed flow system model used for creating biofilm on miniscrews and tooth roots. After inoculation with *Streptococcus mutans,* the miniscrew and tooth root samples were placed in soft agar gel inside a gradient culture container maintained in a 37 ^°^C incubator. A gas pack was used to create am anaerobic environment inside the incubator. BHI medium with 8% sucrose was perfused into the container with a pump flow rate of 0.5 ml/min., which then drained into the waste beaker. BHI = brain heart infusion

The bacteria suspension was then removed, and the upper chamber was washed with 5 ml of BHI medium. The modified flow system model was used to perfuse BHI medium with 8% sucrose (Sigma‐Aldrich, Inc., St. Louis, Missouri, USA) at a flow rate 0.5 ml/min during the biofilm creation (Figure 1; Crusz et al., 2012). Anaerobic condition was induced in the incubator by using an anaerobic gas pack (ANAEROGEN: ThermoFisher SCIENTIFIC Ltd., Waltham, USA). The *S*. *mutans* biofilms were grown for 24 and 48 hr before samples were harvested.

### 2.4 Confocal laser scanning microscopy (CLSM)

The biofilm samples on miniscrews and tooth roots were placed in 24‐well plates and gently washed with 1 ml of phosphate‐buffered saline. Then was stained with a LIVE/DEAD BacLight bacteria viability kit (ThermoFisher SCIENTIFIC Ltd., Waltham, USA) for 30 min. The biofilms were then observed under CLSM at a 4× magnification (excitation wave length 488 nm; Carvalho et al., 2012). The photos were taken from all parts of the sample to create a whole composite of the miniscrew or tooth root from which biofilm migration distances were measured.

### 2.5 Scanning electron microscopy (SEM)

The biofilm‐laden samples were washed gently with 1 ml phosphate‐buffered saline in 24‐well plates. The sample was dehydrated carried out by soaking them in serial ethanol solutions of increasing concentration: 70%, 85%, 95%, and 100% for 30 min each.

The samples were fixed by incubating in 2.5% glutaraldehyde for 2 hr before air‐drying in a chamber overnight. After complete drying, the samples were coated with platinum alloy before imaging (Asahi et al., 2015). The SEM images were randomly taken with three images for each sample at magnification 350× and 1,000×. The images were analyzed descriptively and compared between each group.

### 2.6 Statistical analysis

One way analysis of variance analysis and multiple analysis was used to validate the difference in biofilm migration distance between sample groups: 24‐hr biofilms on miniscrews with ihPDL cells, tooth roots with ihPDL cells, miniscrews without ihPDL cells, tooth roots without ihPDL cells, 48‐hr biofilm on miniscrews with ihPDL cells, tooth roots with ihPDL cells, miniscrews without ihPDL cells, and tooth roots without ihPDL cells.

## 3 RESULTS

### 3.1 CLSM

The 24‐and 48‐hr biofilm growth on miniscrews and tooth roots were tracked under CLSM at 4× magnification. The biofilm was stained with Live/Dead staining to observe the biofilm formation.

**Figure 2:**
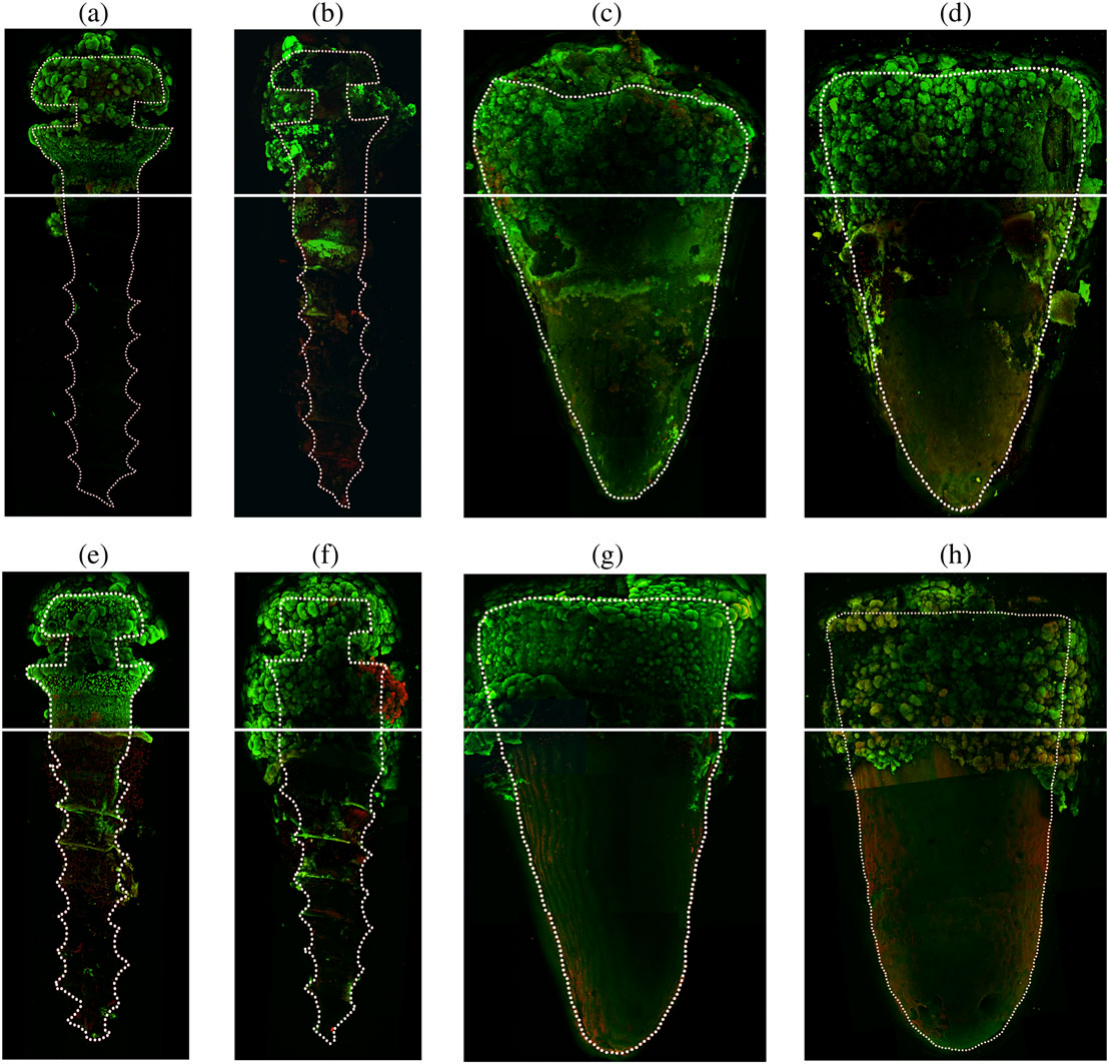
Representative confocal laser scanning images at 4× magnification. The *Streptococcus mutans* biofilm morphology formed into a dome‐shape distribution on miniscrews/tooth root surfaces above the gel level (interface = white line). (a) 24‐Hr biofilm grown on miniscrews with ihPDL cells in the system, (b) 48‐hr biofilm grown on miniscrews with ihPDL cells in the system, (c) 24‐hr biofilm grown on tooth roots with ihPDL cells in the system, (d) 48‐hr biofilm grown on tooth roots with ihPDL cells in the system, (e) 24‐hr biofilm grown on miniscrews without ihPDL cells in the system, (f) 48‐hr biofilm grown on miniscrews without ihPDL cells in the system, (g) 24‐hr biofilm grown on tooth roots without ihPDL cells in the system, (h) 48‐hr biofilm grown on tooth roots without ihPDL cells in the system. ihPDL = immortalized human periodontal ligament

The distribution of the dome‐shaped biofilm could be observed on the surface of titanium miniscrews and tooth roots. At 24 hr, the dome‐shaped biofilms formed on miniscrew were less, both with and without PDL cells, compared with biofilms grown on the tooth roots (Figure 2a,c,e,g). At 48 hr, the biofilm appearance were similar in both the miniscrew surfaces and the tooth roots either with or without the existence of PDL cells (Figure 2b,d,f,h).

### 3.2 SEM

**Figure 3:**
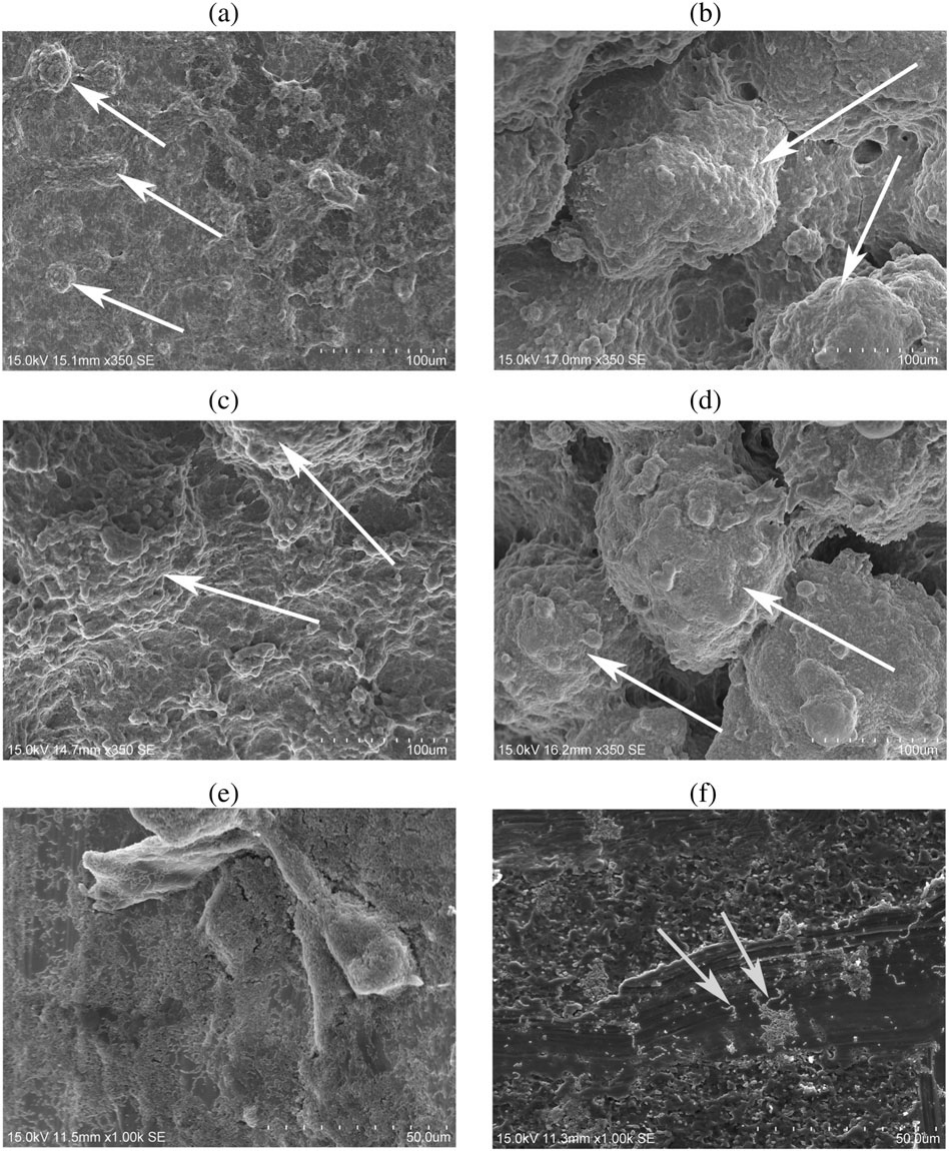
Representative scanning electron images of *Streptococcus mutans* grown on titanium miniscrews and tooth roots surfaces with ihPDL cells in the system. (a) The *S*. *mutans* biofilm grown on miniscrews at 24 hr at a magnification of 350×; (b) the *S*. *mutans* biofilm grown on miniscrews at 48 hr at a magnification of 350x; (C) the *S. mutans* biofilm grown tooth roots at 24 hours at a magnification 350×; (d) the *S*. *mutans* biofilm grown on tooth roots at 48 hr; (e) the apical margin of *S*. *mutans* biofilm grown on miniscrews at 48 hr at a magnification of 1,000×; and (f) the *S*. *mutans* colonies separately located from the biofilm at magnification 1,000×

SEM images at surfaces show details of the biofilm morphology in higher magnification (350× and 1,000×). At 24 hr, the observed biofilms on tooth root samples have more prominence dome shape appearance than those grown on miniscrews (Figure 3a,c). However, the 48‐hr biofilm grown on both miniscrews and tooth roots have similar appearances, displaying a prominent dome‐shape and the *S*. *mutans* cell shape was barely be seen within view (Figure 3b,d).

Below the soft agar gel level, of the *S*. *mutans* biofilm showed a thin flat with an irregular shape, in both the 24‐ and 48‐hr biofilm groups, on miniscrews or tooth roots surfaces, with and without PDL cells include within the system. At the margin, of the apical side of the biofilm under the gel level, the *S*. *mutans* cells are clustered into a gelatinous matrix and forming a biofilm, however, the exopolysaccharide are barely observed on the margin of the biofilm (Figure 3e). *S*. *mutans* colonies found separately from the biofilm show clearly visible of bacteria cell shape: rounded or rod‐like while making a long chains (Figure 3f).

### 3.3 Bacterial biofilm migration

The bacterial biofilm migration distances were measured from confocal laser scanning images. Specifically, this was done by measuring the vertical distance from the interface of the gel level to the most apical biofilm margin found.

**Figure 4:**
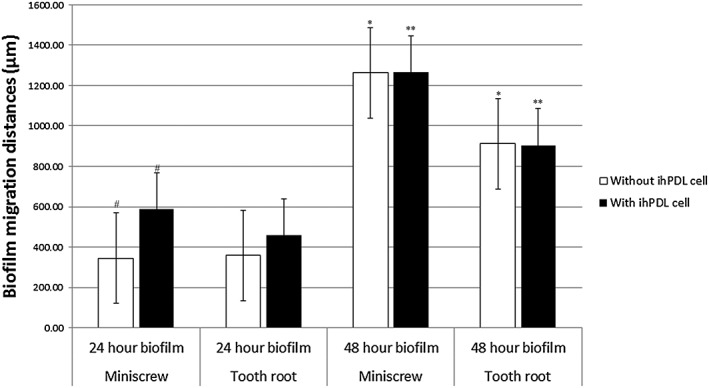
Representative migration distance of *Streptococcus mutans* biofilm on a titanium miniscrews and tooth root surfaces at 24 and 48 hr of biofilm growth with and without ihPDL cells (*n* = 6 for each group). *, ** = significant difference at *p* < .001, # = significant difference at *p* < .05. ihPDL = immortalized human periodontal ligament

The results show that there are no significant differences in biofilm migration between pure titanium miniscrews and tooth roots at 24 hr. However, there are significant differences in biofilm migration between miniscrews with ihPDL cells that is higher than those without ihPDL cells (*p* < .05; Figure 4).

The 48‐hr biofilm migration showed no significant differences in migratory distances in miniscrew group with ihPDL cells and miniscrew group without ihPDL cells (*p* > .05). The same manner also happens to the tooth root group between with and without ihPDL cells (*p* > .05). There were significant higher in the biofilm migration rates in miniscrews group both with and without ihPDL cells compare with tooth roots group with and without ihPDL cells in the system (*p* < .001; Figure 4).

## 4 DISCUSSION

Periodontal and peri‐implant bacterial biofilms are a major source of bacterial infection causing inflammation associated with periodontitis and peri‐implantitis diseases. The bacterial biofilm behavior is an important factor modulates the virulence of the bacteria (Hasan & Palmer, 2014). In this study, the results showed that among tooth root surfaces, a thicker biofilm developed, in the first 24 hr, than that observed on miniscrews. This might be explained by the increased surface roughness of the tooth root compared with the polished surface of titanium miniscrews. Surface roughness is a key factor in biofilm for developing biofilms. The higher surface roughness facilitates easier bacterial attachment to the surface and initiates formation of biofilm (Dhir, 2013; Souza et al., 2016; Subramani et al., 2009).

At 48 hr, the miniscrew and tooth root biofilms observed by SEM share similar appearances of dome‐shape protrusions dominating the biofilm affected surfaces. The dome‐shape structures were a common characteristic morphology ascribed to the *S*. *mutans* bacterial biofilms. As when the biofilms matured, the dome‐shaped structures also grew larger with increased thicknesses (Koo et al., 2010).

Confocal laser scanning images also confirmed the bacterial biofilm pattern viewed under the SEM. The *S*. *mutans* biofilm observed at 24 hr on the tooth root surface were more compact and emitted a stronger fluorescence than those on miniscrew surfaces, regardless of the presence of ihPDL cells in the system. Although biofilm structure morphologies are quite similar in what between tooth root and miniscrew surfaces, biofilm formation was more rapidly established on the tooth root surface within the first 24 hr than on titanium miniscrew surfaces. This corresponded to the SEM image results.

Biofilms were observed to migrate downward along the surface of miniscrews and tooth root since first 24 hr. However, as observed after 24 hr, the morphology of the biofilm located under the gel interface, manifested as flattened, irregular patterning was significantly different from biofilm above the gel level.

*Streptococcus mutans* is a nonmotile bacteria meaning that they cannot independently move and relocate to new environments and form colonies. This can only occur if there is a free‐flow of saliva from the oral cavity (*in vivo*) or media from the culture chamber (*in vitro*; Daboor, Masood, Al‐Azab, & Nori, 2015). In this *in vitro* system, flow of media above the gel level occurs at a rate of 0.5 ml/min. Thus, below the gel level, biofilm formation occurs as a result of bacterial migration caused by the pushing force from bacterial proliferation and expansion.

The biofilm below the gel interface is patterned into a flat and irregular structure probably due to the restricted space for the bacterial biofilm to expand into (Figure 3e). Bacteria located at the gel margin pushes itself downward into the only available free‐space, generates a biofilm that cannot form its normal morphology.

The alteration of biofilms morphology could also be caused by a lack of nutrient penetration further below the gel margin and interface. *S*. *mutans* mainly create water‐insoluble glucan that will form a semisolid solid polysaccharide to cover the bacteria cell (Koo et al., 2010; Mattos‐Graner et al., 2000); which corresponds to the result from SEM images, in which bacteria cells can be hardly seen (Figure 3a–d). The extracellular polysaccharide forms the gelatinous bulk structure of the biofilm and protects the bacteria from harmful environmental conditions (Bowen & Koo, 2011; Krzyściak, Jurczak, Kościelniak, Bystrowska, & Skalniak, 2014; Welin‐Neilands & Svensäter, 2007). Nutrients are lacking below the gel interface with the media, and this starves *S*. *mutans* bacteria resulting in a poor biofilm assemblage and a dysfunctional morphology.

The biofilm migration was measured according to the longest distance the biofilm travelled from the gel interface as observed in confocal scanning images. The starting point for measurement was taken at the margin of the gel interface, which was located at 6,000 nm from the tip of the miniscrew and tooth root. Biofilm migration length was measured in relation to a continuous line of green fluorescence from stained bacteria. Green fluorescence traces that were discontinuous were not counted as true biofilms migration.

The data clearly showed that the migratory distance (length from the gel interface) of the biofilm along miniscrews versus tooth roots groups, at 24 hr, was not significantly different in the presence or absence of PDL cells (Figure 4). This contradicted the data from SEM showing that the initial biofilm forming on the tooth root surface was faster than that on the titanium miniscrew surface, at 24 hr.

Interestingly at 24 hr of culture, the biofilm developed on the miniscrews in the presence of PDLs and migrated faster than those developed on miniscrews without the PDL cells being present. Thus, there was only a difference in migration distances among miniscrews but not in the tooth root group during 24 hr. It indicates that in the early stage of bacteria formation, the PDL cell may attract bacterial biofilm grown on miniscrew to migrate toward itself. However, there are still controversies whether PDL cells have an influence on biofilm migration or not because *S*. *mutans* is not a principal participant in periodontal bacterial species pathogenesis and does not interact with PDL cells in the same manner as periodontal related species—*Aggregatibacter actinomycetemcomitans*—have a direct function through the lipopolysaccharide/toll‐like receptors nor do they possess virulent factors to attach and host cell same as *Porphyromonas gingivalis* (Riep et al., 2009; Sakanaka, Takeuchi, Kuboniwa, & Amano, 2016; Sun, Shu, Li, & Zhang, 2010).

Furthermore, the flow system model used in this study allowed the PDL cells to remain in anaerobic conditions, which does alter the cell stress response to the environment through the hypoxia inducible factor expression and might affect the cell response to the bacteria (Wang, Chen, & Leung, 2017).

Therefore, the model developed for this study has some flaws in proving to us whether PDL cells have an effect on bacterial biofilm migration capabilities or not as the contradictory factors state above. Further investigation is needed.

The migration rates of biofilms growing on miniscrew surfaces are significantly faster than the biofilms growing on the tooth root surface at 48 hr, whether there are ihPDL cells in the system or not (Figure 4). However, there are no significant differences in migration rates between miniscrews with ihPDL cells in the system and those without ihPDL cells in the system at 48‐hr biofilm measurements (*p* > .05).

It may be assumed that at the later stage of biofilm formation, the influence of ihPDL cells is low strength, or the condition of ihPDL cells is poor due to the anaerobic condition (Wang et al., 2017). The results from the 48‐hr biofilm indicate that the bacterial biofilm behave differently on various materials surfaces with different chemical properties, including their migration rates (Dhir, 2013; Yoshida et al., 2013).

*Streptococcus mutans* is a core bacterial species during the early stage of biofilm formation. The *S*. *mutans* bacterial biofilm formation and migration might represent the biofilm migration and spreading on solid surfaces in the oral cavity before the microbiota shifts to late colonizer and starts causing inflammation, leading to periodontitis or peri‐implantitis (Berezow & Darveau, 2011).

In summary, the *S*. *mutans* biofilm growth on titanium miniscrews and natural tooth roots does not produce any differences in morphology. However, the biofilm initially developed on the tooth roots faster than on the miniscrews. The biofilm migration rate is clearly faster on the titanium miniscrew surfaces compared with the natural tooth root.

The flow system model in this study could not clearly identify the role of PDL cell involvement in biofilm migration due to certain limitations of the anaerobic conditions and the interactions between bacteria and PDL cells.

Further experiments should continue to study the periodontal pathogenic bacterial biofilms with direct interactions with the periodontal tissue cell. Moreover, the flow model system used in this experiment shall be developed to match the anaerobic conditions needed for bacteria concurrently with the aerobic conditions for the PDL cells. Furthermore, the media for growing biofilm in the current study contained sugar, which was meant to achieve the supragingival condition where *S*. *mutans* generally located. Therefore, to achieve the subgingival condition, sugar‐free media should be used to culture the biofilm.

## CONFLICT OF INTEREST

The authors declare no potential conflicts of interest with respect to publication and the authorship of this article.

## References

[cre2101-bib-0001] Asahi, Y. , Miura, J. , Tsuda, T. , Kuwabata, S. , Tsunashima, K. , Noiri, Y. , … Hayashi, M. (2015). Simple observation of Streptococcus mutans biofilm by scanning electron microscopy using ionic liquids. AMB Express, 5(1), 6. 25642403 10.1186/s13568-015-0097-4 PMC4305086

[cre2101-bib-0002] Berechet, C. A. , Ionaşcu, A. M. , Sîrbu, V. , & Sîrbu, I. (2013). Peri‐implantitis versus periodontitis‐similarities and differences. Literature review. Romanian Journal of Stomatology, 59(1), 32–37.

[cre2101-bib-0003] Berezow, A. B. , & Darveau, R. P. (2011). Microbial shift and periodontitis. Periodontology 2000, 55(1), 36–47. 21134227 10.1111/j.1600-0757.2010.00350.x PMC3058494

[cre2101-bib-0004] Berglundh, T. , Zitzmann, N. U. , & Donati, M. (2011). Are peri‐implantitis lesions different from periodontitis lesions? Journal of Clinical Periodontology, 38(s11), 188–202. 21323715 10.1111/j.1600-051X.2010.01672.x

[cre2101-bib-0005] Bowen, W. , & Koo, H. (2011). Biology of Streptococcus mutans‐derived glucosyltransferases: Role in extracellular matrix formation of cariogenic biofilms. Caries Research, 45(1), 69–86. 10.1159/000324598 PMC3068567 21346355

[cre2101-bib-0006] Busscher, H. J. , Rinastiti, M. , Siswomihardjo, W. , & Van der Mei, H. C. (2010). Biofilm formation on dental restorative and implant materials. Journal of Dental Research, 89(7), 657–665. 20448246 10.1177/0022034510368644

[cre2101-bib-0012] Carvalho, F. G. D. , Puppin‐Rontani, R. M. , Fúcio, S. B. P. D. , Negrini, T. D. C. , Carlo, H. L. , & Garcia‐Godoy, F. (2012). Analysis by confocal laser scanning microscopy of the MDPB bactericidal effect on S. mutans biofilm CLSM analysis of MDPB bactericidal effect on biofilm. Journal of Applied Oral Science, 20(5), 568–575. 23138745 10.1590/S1678-77572012000500013 PMC3881786

[cre2101-bib-0007] Crusz, S. A. , Popat, R. , Rybtke, M. T. , Cámara, M. , Givskov, M. , Tolker‐Nielsen, T. , … Williams, P. (2012). Bursting the bubble on bacterial biofilms: A flow cell methodology. Biofouling, 28(8), 835–842. 22877233 10.1080/08927014.2012.716044 PMC3438488

[cre2101-bib-0008] Daboor, S. M. , Masood, F. S. S. , Al‐Azab, M. S. , & Nori, E. E. (2015). A review on streptococcus mutans with its diseases dental caries, dental plaque and endocarditis. Indian Journal of Microbiology Research, 2(2), 76–82.

[cre2101-bib-0009] Dani, S. , Prabhu, A. , Chaitra, K. R. , Desai, N. C. , Patil, S. R. , & Rajeev, R. (2016). Assessment of *Streptococcus mutans* in healthy versus gingivitis and chronic periodontitis: A clinico‐microbiological study. Contemporary clinical dentistry, 7(4), 529. 27994423 10.4103/0976-237X.194114 PMC5141670

[cre2101-bib-0010] Dhir, S. (2013). Biofilm and dental implant: The microbial link. Journal of Indian Society of Periodontology, 17(1), 5. 23633764 10.4103/0972-124X.107466 PMC3636945

[cre2101-bib-0011] Dhir, S. , Mahesh, L. , Kurtzman, G. M. , & Vandana, K. L. (2013). Peri‐implant and periodontal tissues: A review of differences and similarities. Compendium of continuing education in dentistry (Jamesburg, NJ: 1995), 34(7):e69–75. 24428439

[cre2101-bib-0013] Hasan, A. , & Palmer, R. M. (2014). A clinical guide to periodontology: Pathology of periodontal disease. British Dental Journal, 216(8), 457–461. 24762896 10.1038/sj.bdj.2014.299

[cre2101-bib-0014] Horibata, S. , Vo, T. V. , Subramanian, V. , Thompson, P. R. , & Coonrod, S. A. (2015). Coonrod SA. Utilization of the soft agar colony formation assay to identify inhibitors of tumorigenicity in breast cancer cells. Journal of visualized experiments: JoVE, (99). 10.3791/52727 PMC4542786 26067809

[cre2101-bib-0015] Koo, H. , Xiao, J. , Klein, M. I. , & Jeon, J. G. (2010). Exopolysaccharides produced by *Streptococcus mutans* glucosyltransferases modulate the establishment of microcolonies within multispecies biofilms. Journal of Bacteriology, 192(12), 3024–3032. 20233920 10.1128/JB.01649-09 PMC2901689

[cre2101-bib-0016] Krzyściak, W. , Jurczak, A. , Kościelniak, D. , Bystrowska, B. , & Skalniak, A. (2014). The virulence of Streptococcus mutans and the ability to form biofilms. European Journal of Clinical Microbiology & Infectious Diseases, 33(4), 499–515. 24154653 10.1007/s10096-013-1993-7 PMC3953549

[cre2101-bib-0017] Marchesan, J. T. , Scanlon, C. S. , Soehren, S. , Matsuo, M. , & Kapila, Y. L. (2011). Implications of cultured periodontal ligament cells for the clinical and experimental setting: A review. Archives of Oral Biology, 56(10), 933–943. 21470594 10.1016/j.archoralbio.2011.03.003 PMC3132241

[cre2101-bib-0018] Mattos‐Graner, R. O. , Smith, D. J. , King, W. F. , & Mayer, M. P. A. (2000). Water‐insoluble glucan synthesis by mutans streptococcal strains correlates with caries incidence in 12‐to 30‐month‐old children. Journal of Dental Research, 79(6), 1371–1377. 10890715 10.1177/00220345000790060401

[cre2101-bib-0019] Palomo, L. , & Terézhalmy, G. T. (2014). Peri‐implant disease: Pathogenesis, risk factors, diagnosis, prevention and treatment. Provider, 501, 211886.

[cre2101-bib-0020] Patel, S. S. , Aruni, W. , Inceoglu, S. , Akpolat, Y. T. , Botimer, G. D. , Cheng, W. K. , & Danisa, O. A. (2016). A comparison of Staphylococcus aureus biofilm formation on cobalt‐chrome and titanium‐alloy spinal implants. Journal of Clinical Neuroscience, 31, 219–223. 27396378 10.1016/j.jocn.2016.03.013

[cre2101-bib-0021] Riep, B. , Edesi‐Neuß, L. , Claessen, F. , Skarabis, H. , Ehmke, B. , Flemmig, T. F. , … Moter, A. (2009). Are putative periodontal pathogens reliable diagnostic markers? Journal of Clinical Microbiology, 47(6), 1705–1711. 19386852 10.1128/JCM.01387-08 PMC2691128

[cre2101-bib-0022] Sakanaka, A. , Takeuchi, H. , Kuboniwa, M. , & Amano, A. (2016). Dual lifestyle of Porphyromonas gingivalis in biofilm and gingival cells. Microbial Pathogenesis, 94, 42–47. 26456558 10.1016/j.micpath.2015.10.003

[cre2101-bib-0023] Souza, J. , Mota, R. R. , Sordi, M. B. , Passoni, B. B. , Benfatti, C. A. , & Magini, R. S. (2016). Biofilm formation on different materials used in oral rehabilitation. Brazilian Dental Journal, 27(2), 141–147. 27058375 10.1590/0103-6440201600625

[cre2101-bib-0024] Subramani, K. , Jung, R. E. , Molenberg, A. , & Hämmerle, C. H. (2009). Biofilm on dental implants: A review of the literature. International Journal of Oral & Maxillofacial Implants, 24, 616–626. 19885401

[cre2101-bib-0025] Sun, Y. , Shu, R. , Li, C. L. , & Zhang, M. Z. (2010). Gram‐negative periodontal bacteria induce the activation of toll‐like receptors 2 and 4, and cytokine production in human periodontal ligament cells. Journal of Periodontology, 81(10), 1488–1496. 20528699 10.1902/jop.2010.100004

[cre2101-bib-0026] Wang, X. X ., Chen, Y ., Leung, W. K . (2017). Role of the hypoxia‐inducible factor in periodontal inflammation, Hypoxia and human diseases. InTech, Available from: https://www.intechopen.com/books/hypoxia-and-human-diseases/role-of-the-hypoxia-inducible-factor-in-periodontal-inflammation

[cre2101-bib-0027] Welin‐Neilands, J. , & Svensäter, G. (2007). Acid tolerance of biofilm cells of Streptococcus mutans. Applied and Environmental Microbiology, 73(17), 5633–5638. 17630302 10.1128/AEM.01049-07 PMC2042095

[cre2101-bib-0028] Yoshida, E. , Imai, S. , Hanada, N. , & Hayakawa, T. (2013). Biofilm bormation on titanium and hydroxyapatite surface using artificial mouth system. Journal of Hard Tissue Biology, 22(4), 419–424.

